# Identification of the molecular function of tripartite motif containing 58 in human lung cancer

**DOI:** 10.3892/ol.2021.12946

**Published:** 2021-07-28

**Authors:** Yuan-Xiang Shi

**Affiliations:** Institute of Clinical Medicine, Hunan Provincial People's Hospital, The First Affiliated Hospital of Hunan Normal University, Changsha, Hunan 410005, P.R. China

**Keywords:** lung cancer, early diagnosis, malignant phenotype, tripartite motif containing 58

## Abstract

Lung cancer is a major public health problem worldwide, with a high associated incidence and mortality. In the present study, novel epigenetic signatures were identified through genome-wide DNA methylation microarrays. The results revealed that tripartite motif containing 58 (TRIM58), a potential tumor suppressor gene exhibited high methylation and low expression in lung cancer tissue samples compared with normal tissues. Receiver operating characteristic curve analysis demonstrated that TRIM58 may be a promising early diagnostic indicator of lung cancer. In addition, the present study analyzed the role of TRIM58 in tumorigenesis and development in lung cancer A549 cells. Wound healing assay and transwell migration assay were used to investigate cell migration, and flow cytometry analysis was used to detect apoptosis. Silencing TRIM58 accelerated the proliferation and migration of lung cancer cells. In contrast, the overexpression of TRIM58 significantly inhibited the proliferation and migration of lung cancer cells and promoted apoptosis. Gene set enrichment analysis revealed that TRIM58 expression was negatively correlated with MYC targets, G_2_M checkpoints and the mTORC1 signaling pathway. These results of the present study suggested that TRIM58, a potential tumor suppressor gene may serve as a novel diagnostic biomarker and therapeutic target in human lung cancer.

## Introduction

According to the Cancer Statistics report published by the American Cancer Society in 2021, lung cancer is the leading cause of death in both men and women, accounting for 22% of all cancer deaths (1). In China, lung cancer ranks first among malignant tumors in incidence and mortality and has become an important disease that endangers public health and affects people's quality of life (2). Lung cancer lacks obvious clinical symptoms in the early stage, and at the same time lacks effective early screening methods, the 5-year survival rate of lung cancer patients is less than 15% (3,4). In addition, metastasis and chemoresistance are important causes of the high mortality in lung cancer (5,6). Hence, the identification of specific molecular markers and the exploration of new effective drug targets for the early diagnosis and treatment of lung cancer are urgently needed and have extremely important scientific research significance and clinical application prospects (7).

Epigenetic events are important in all aspects of biology, and numerous studies have shown that they serve key roles in carcinogenesis and tumor progression (8–10). Various mechanisms contribute to the occurrence of lung cancer, including DNA methylation. However, the specific regulatory mechanisms have not been fully elucidated (11). DNA methylation is an important epigenetic regulation and abnormal methylation can affect gene expression (12,13). Molecular markers of DNA methylation for the early diagnosis and prognosis prediction of tumors and tumor-targeting drugs based on epigenetics have been widely studied, for example, the application of O-6-methylguanine-DNA methyltransferase (MGMT) DNA methylation in molecular diagnosis of glioma (14–16). The identification of DNA methylation profiles in tumors has laid the foundation for the discovery of new tumor therapeutic targets.

Tripartite motif containing 58 (TRIM58) is a member of the tripartite motif (TRIM) family (17). The TRIM protein family is a conserved protein family that plays important roles in signal transduction, innate immunity, autophagy, tumors and other functions (18,19). For instance, TRIM67 inhibits the occurrence and progression of colorectal cancer by activating the p53 signaling pathway (20). The TRIM family is characterized by 3 domains (from the N-terminus to the C-end): the RING (Really Interesting New Gene) -finger domain, one or two B-boxes, and one coiled-coil domain (21). Currently, more than 80 TRIM proteins have been found in humans, most of which have the function of E3 ubiquitin ligase and regulate cell transcription, proliferation and apoptosis through the ubiquitination of target molecules, thus participating in various physiological and pathological processes in the body, such as developmental disorders, viral infections and cancer (22,23). According to the features of the domains, the TRIM family is divided into 11 subfamilies and TRIM58 is a member of the C-IV-1 subfamily (C-I to C-XI) (24).

The main subtypes of lung cancer are lung adenocarcinoma (LUAD) and lung squamous cell carcinoma (LUSC) (25). In our previous study, we collected clinical samples of LUSC for genome-wide DNA methylation analysis and identified many new epigenetic signatures (26). In the present study 3 methylation microarray datasets (GSE63384, GSE62948 and GSE32861) of LUAD from the Gene Expression Omnibus (GEO) database were collected for the integrated analysis of large samples. Integrating the results of high-throughput screening, focusing on TRIM58, which was hypermethylated and downregulated in lung cancer. Notably, functional studies performed demonstrated that overexpression of TRIM58 inhibited cell proliferation and migration and promoted cell apoptosis. These findings suggest that TRIM58 serves a critical role in the malignant phenotype of lung cancer.

## Materials and methods

### 

#### DNA methylation datasets of lung cancer

The Gene Expression Omnibus (GEO) datasets (GSE63384, GSE62948 and GSE32861) were all based on the GPL8490 platform (http://www.ncbi.nlm.nih.gov/geo) (27–29). The 3 datasets selected all comprised of paired samples consisting of tumor and corresponding NTL tissues. The GSE63384 dataset included 35 stage I LUAD tissues and 35 NTL tissues; GSE62948 included 28 LUAD tissues and 28 NTL tissues and GSE32861 contained 59 LUAD tissues and 59 NTL tissues. Subsequently, these 3 datasets were used for ROC analysis.

#### Genome-wide DNA methylation analysis

Genome-wide DNA methylation analysis using the R package version 4.2 (http://www.r-project.org/) (30). The linear models for microarray data (LIMMA) package (v.3.48.0) in Bioconductor was used for data processing (31). The Benjamini-Hochberg procedure in R package was used to calculate the adjusted P-values (32). Probes with a adjusted P<0.05 and an absolute β difference ≥ 0.2 were considered differentially methylated genes (DMGs).

#### The Cancer Genome Atlas (TCGA) data and validation

Validation datasets were extracted from the data portal of TCGA (http://tcga-data.nci.nih.gov) (33). TCGA DNA methylation dataset: A total of 372 LUSC samples with 43 corresponding NTL samples and 460 LUAD samples with 32 corresponding NTL samples were used for performing independent DNA methylation verification. TCGA mRNA expression dataset: including 502 LUSC samples corresponding to 51 NTL samples, and 571 LUAD samples corresponding to 58 NTL samples for mRNA expression detection. In addition, the MethHC browser (http://methhc.mbc.nctu.edu.tw/php/index.php) was used to analyze the correlation between DNA methylation and mRNA expression (34).

#### Cell culture and transfection

A549, a human lung adenocarcinoma cell line was purchased from the Chinese Academy of Sciences and cultured at 37°C with 5% CO_2_ in RPMI-1640 medium (Invitrogen; Thermo Fisher Scientific Inc.) containing 10% fetal bovine serum (FBS, Gibco; Thermo Fisher Scientific Inc.).

To investigate the molecular functions of TRIM58, specific small interfering (si) RNA and overexpression vectors of TRIM58 were constructed. Transfection was performed 24 h after the cells were plated. TRIM58-siRNA was synthesized by Guangzhou RiboBio Co., Ltd. and the target sequence was 5′-GGACTATGAAGCCGGTGAA-3′. For the scrambled siRNA used as the negative control (NC) (Guangzhou RiboBio Co., Ltd.). The final siRNA (TRIM58-siRNA or scrambled siRNA) concentration was adjusted to 50nM and transfected with Lipofectamine^®^ RNAiMAX (Invitrogen; Thermo Fisher Scientific Inc.). pCDNA3.1-TRIM58 vector was synthesized by Shanghai GeneChem Co., Ltd. and empty pcDNA3.1 vector was used as the negative control. The final pcDNA3.1 vector concentration was adjusted to 1μg and transfected with Lipofectamine 2000 (Invitrogen; Thermo Fisher Scientific Inc.). After transfection for 48 h at 37°C, cell migration was detected and cells were collected for RNA and protein extraction.

#### RNA extraction and reverse transcription-quantitative (RT-q) PCR

A549 cells were collected. TRIzol^®^ reagent (Invitrogen; Thermo Fisher Scientific Inc.) was used to extract total RNA. The PrimeScript™ RT reagent kit (Takara Bio, Inc.) was used for reverse transcription and the standard SYBR Green PCR kit (cat. no. RR091A; Takara Bio, Inc.) was used for RT-qPCR according to the manufacturer's protocol. The PCR thermocycling conditions were as follows: initial denaturation at 95°C for 10 min, followed by 40 cycles at 95°C for 15 sec and 60°C for 60 sec. GAPDH was used as the internal reference gene and the data were calculated using the 2^−∆∆CT^ method (35). The primer sequences used were as follows: GAPDH, forward 5′-GGAAGCTTGTCATCAATGGAAATC-3′ and reverse, 5′-TGATGACCCTTTTGGCTCCC-3′; TRIM58 forward, 5′-ATGAGGAAAGAGTTGGAGGACG-3′ and reverse, 5′-AGCCACGATGCTTCTCAAACTC-3′.

#### Western blotting

A549 cells were collected and protein was extracted using RIPA lysis buffer (Abcam). The bbicinchoninic acid (BCA) kits were used to detect protein concentrations. The total protein (40 µg/lane) was separated by 10% SDS-PAGE and transferred to nitrocellulose membrane. Subsequently, the membrane was incubated in a blocking solution (5% skimmed milk) for 2 h at room temperature. The primary antibody was incubated with the samples at 4°C overnight and then the secondary antibody was incubated with the samples at room temperature for 2 h. The primary antibodies used were as follows: GAPDH [cat. no. abs132004; 1: 3000; Absin (Shanghai) Biotechnology Co. Ltd.] and TRIM58 [cat. no. abs103739; 1: 1000; Absin (Shanghai) Biotechnology Co. Ltd.]. GAPDH was used as the loading control. Anti-rabbit IgG, HRP-linked Antibody [cat. no. 7074P2; 1: 1000; Cell Signaling Technology Inc.]. ECL luminescence reagent [cat. no. abs920; Absin (Shanghai) Biotechnology Co. Ltd.] was used for protein visualization.

#### Cell proliferation assay

A549 cells were seeded in 96-well plates and cell proliferation was detected with CellTiter 96^®^ AQueous One Solution Cell Proliferation Assay (MTS) (Promega Inc.). The absorbance value was measured at 490 nm.Proliferation was tested every 24 h for 5 consecutive days.

#### Wound healing assay

A549 cells were seeded in 6-well plates (4×10^5^ cells/well). When confluence exceeded 90%, the cell monolayer was damaged with sterile pipette tips. Subsequently, the cells were washed gently and quickly with sterile PBS 3 times and then replaced with RPMI-1640 medium (Invitrogen; Thermo Fisher Scientific Inc.) containing 2% FBS (Gibco; Themo Fisher Scientific Inc.) and incubated at 37°C for 48 h. The cells in the scratch area were observed under a light microscope (magnification, ×100) at 0 and 48 h respectively and the migration distance of cells at each time point was measured manually.

#### Transwell migration assay

Migration experiments were conducted using 24-well transwell chambers with 8-µm aperture (Corning, Inc.). The A549 cells were collected, resuspended with serum-free RPMI-1640 medium and counted. The cell density was adjusted to 4×10^5^ cells/ml. A total of 100 µl cell suspension was inoculated into the upper chamber and 600 µl RPMI-1640 medium containing 20% FBS was added to the lower chamber. Cells were incubated at 37°C for 24 h and stained with 0.1% crystal violet for 20 min at room temperature. Under the light microscope (magnification, ×100), a total of 3 fields were randomly selected for photographing and the number of migrated cells was counted manually.

#### Flow cytometry analysis

A549 cells were centrifuged at 300 × g for 5 min at room temperature and the cell precipitate was collected. Annexin V-FITC/propidium iodide (PI) Apoptosis detection kit (cat. no. V13241; Invitrogen; Thermo Fisher Scientific Inc.) was used to detect cell apoptosis (early apoptosis and late apoptosis) according to the manufacturer's protocol. Apoptotic analysis was implemented using flow cytometry (Cytomics FC500, Beckman Coulter, Inc.). CXP (Beckman Coulter, Inc.) software was used.

#### Gene set enrichment analysis, protein interaction and co-expression analysis

Gene set enrichment analysis (GSEA) (https://www.gsea-msigdb.org/gsea/index.jsp) was performed using mRNA expression data from the TCGA database. Patients were divided into high expression group and low expression group according to the median expression value (LUAD_median_=−1.966, LUSC_median_=−2.457). Protein-Protein Interaction Network analysis was constructed using the STRING database (https://string-db.org/). Co-expression analysis was performed on lung cancer samples from the Oncomine database (https://www.oncomine.org/).

#### Statistical analysis

SPSS version 18.0 (SPSS Inc.) and GraphPad Prism 5.0 software (GraphPad, Inc.) were used for statistical analysis. All data was from 3 experimental replicates and presented as the mean ± SEM. Independent Student's t-test was applied to the comparisons between groups. Spearman correlation method was used to identify the correlation between DNA methylation and mRNA expression. ROC analysis was used to explore the diagnostic value of TRIM58. P<0.05 was considered to indicate statistical significance.

## Results

### 

#### Identification of novel epigenetic signatures in lung cancer

**Figure 1.**
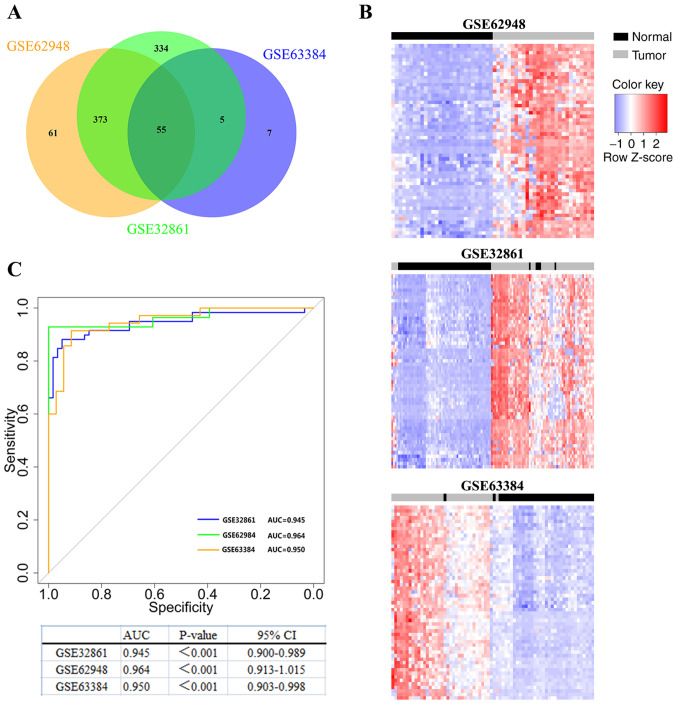
Identification of novel epigenetic signatures in lung cancer. (A) Identify differentially methylated probes through genome-wide DNA methylation analysis. Venn diagram showing that there are 55 common hypermethylated probes between the 3 LUAD datasets (GSE63384, GSE62948 and GSE32861). (B) Two-dimensional cluster analysis was performed on the differentially methylated probes in the 3 datasets. Each row is a probe; each column is a sample. The blue box, the expression level is low; the red box, the expression level is high. (C) Diagnostic value of TRIM58 methylation in lung cancer by ROC curves analysis. The blue line is GSE32861, the green line is GSE62948, and the orange line is GSE63384. ROC, receiver operating characteristic; TRIM58, tripartite motif containing 58; LUAD, lung adenocarcinoma; AUC, area under curve, CI, confidence interval.

In the present study, 3 LUAD methylation profiles (GSE63384, GSE62948, and GSE32861) were collected from the GEO database to identify DMGs between tumors and NTL tissues. The present study focused on probes that were specifically hypermethylated in tumors. The results demonstrated that 67 probes were hypermethylated in GSE63384, 489 probes were hypermethylated in GSE62948 and 767 probes were hypermethylated in GSE32861 (Fig. 1A). Subsequently, an overlapping analysis of these DMGs was conducted and 55 probes that were significantly hypermethylated in the 3 lung cancer datasets were found (Fig. 1A and Table I). Additionally, the 55 probes were analyzed by two-dimensional hierarchical cluster analysis, which could clearly distinguish tumor tissues from NTL tissues (Fig. 1B). These results revealed a series of probes that are hypermethylated in LUAD.

#### TRIM58 serves as a potential diagnostic biomarker for lung cancer

ROC analysis was used to evaluate the diagnostic value of TRIM58 in lung cancer. The area under the curve (AUC) values of the tumor and NTL groups in the TRIM58 analyses were significant for all 3 lung cancer datasets and were as follows: AUC_GSE63384_=0.950 [P <0.001; 95% confidence interval (CI), 0.903–0.998]; AUC_GSE62948_=0.964 (P <0.001; 95% CI, 0.913–1.015) and AUC_GSE32861_=0.945 (P<0.001; 95% CI, 0.900–0.989) (Fig. 1C). The aforementioned results demonstrated that the methylation level of TRIM58 can distinguish tumor tissues from normal tissues and that TRIM58 methylation is a potential marker for the early diagnosis of lung cancer.

#### TRIM58 is coordinately hypermethylated and downregulated in lung cancer

The present study reviewed a large amount of literature on these 55 hypermethylated probes. Among them, TRIM58 is a member of the TRIM family, which is located on chromosome 1 and on CpG islands (36). Previous studies have shown that TRIM protein may serve an important role in tumorigenesis; however, the mechanism by which TRIM58 participates in the regulation of lung cancer remains unclear (17,37).

**Figure 2.**
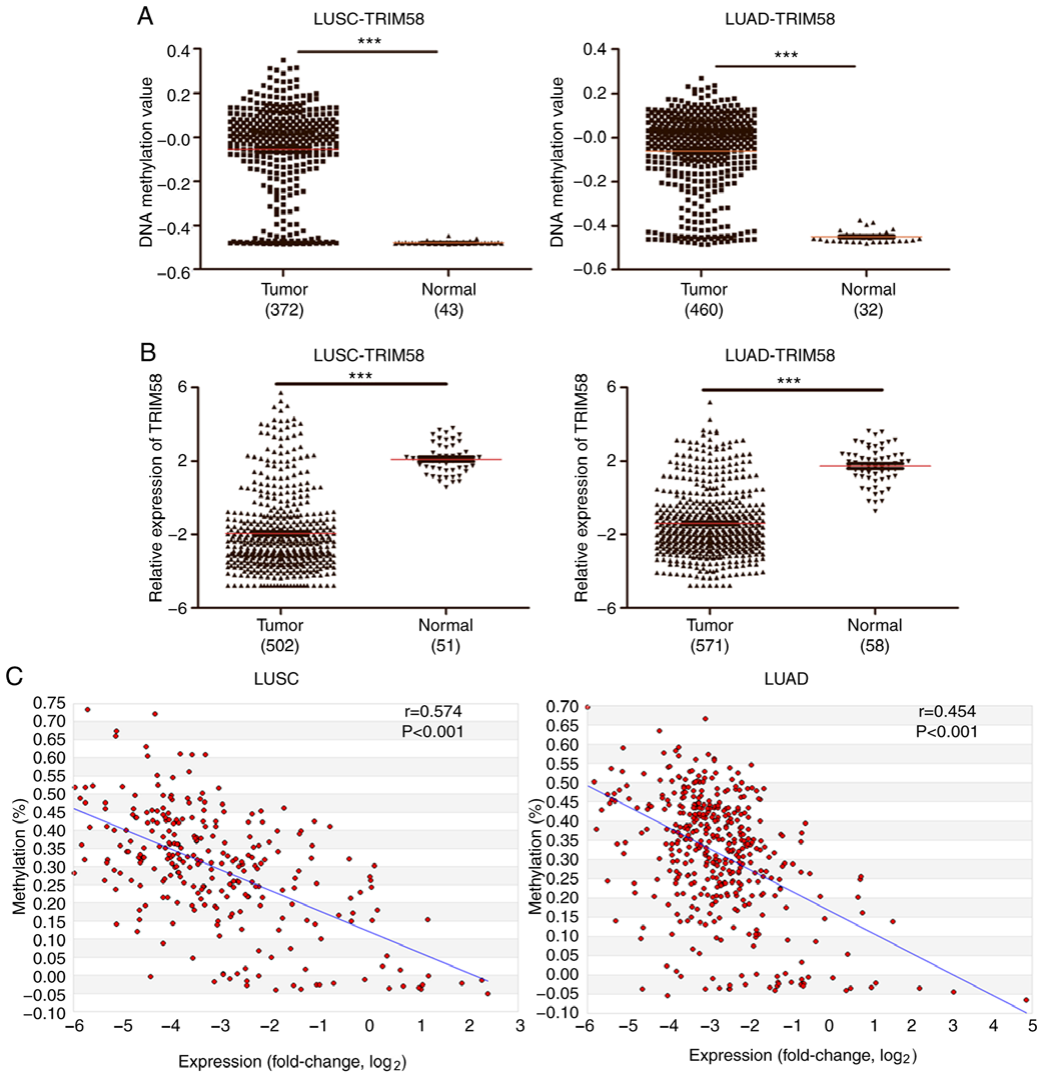
TRIM58 is coordinately hypermethylated and downregulated in lung cancer. (A) Methylation levels of TRIM58 were verified in LUSC and LUAD, respectively. (B) TRIM58 expression was verified in LUSC and LUAD, respectively. (C) Correlation analysis between DNA methylation and gene expression. X-axis, mRNA expression level, Y-axis, DNA methylation level and r, Spearmen correlation coefficient. ***P< 0.001. TRIM58, tripartite motif containing 58; LUAD, lung adenocarcinoma; LUSC, lung squamous cell carcinoma.

Firstly, the results of high-throughput screening were validated using the TCGA datasets. A total of 372 LUSC samples with 43 NTL samples and 460 LUAD samples with 32 NTL samples were used for independent verification. In both, LUSC and LUAD, TRIM58 was hypermethylated in tumor tissues compared to normal tissues (Fig. 2A). In contrast, TRIM58 was downregulated in both LUSC and LUAD compared to normal tissue (Fig. 2B). TRIM58 was coordinately hypermethylated and downregulated in lung tumors compared to normal tissue, indicating that it may be a potential tumor suppressor gene. Subsequently, the correlation between DNA methylation and mRNA expression was analyzed in the MethHC database. Scatter plot analysis revealed that the DNA methylation and mRNA expression levels of TRIM58 were negatively correlated and the Spearman correlation coefficient values were r*_LUSC_* =0.574 and r*_LUAD_* =0.454 (both P<0.001; Fig. 2C). These results suggested that TRIM58 expression may be regulated by epigenetics, DNA methylation in particular.

#### TRIM58 inhibits the malignant phenotypes of lung cancer cells

**Figure 3.**
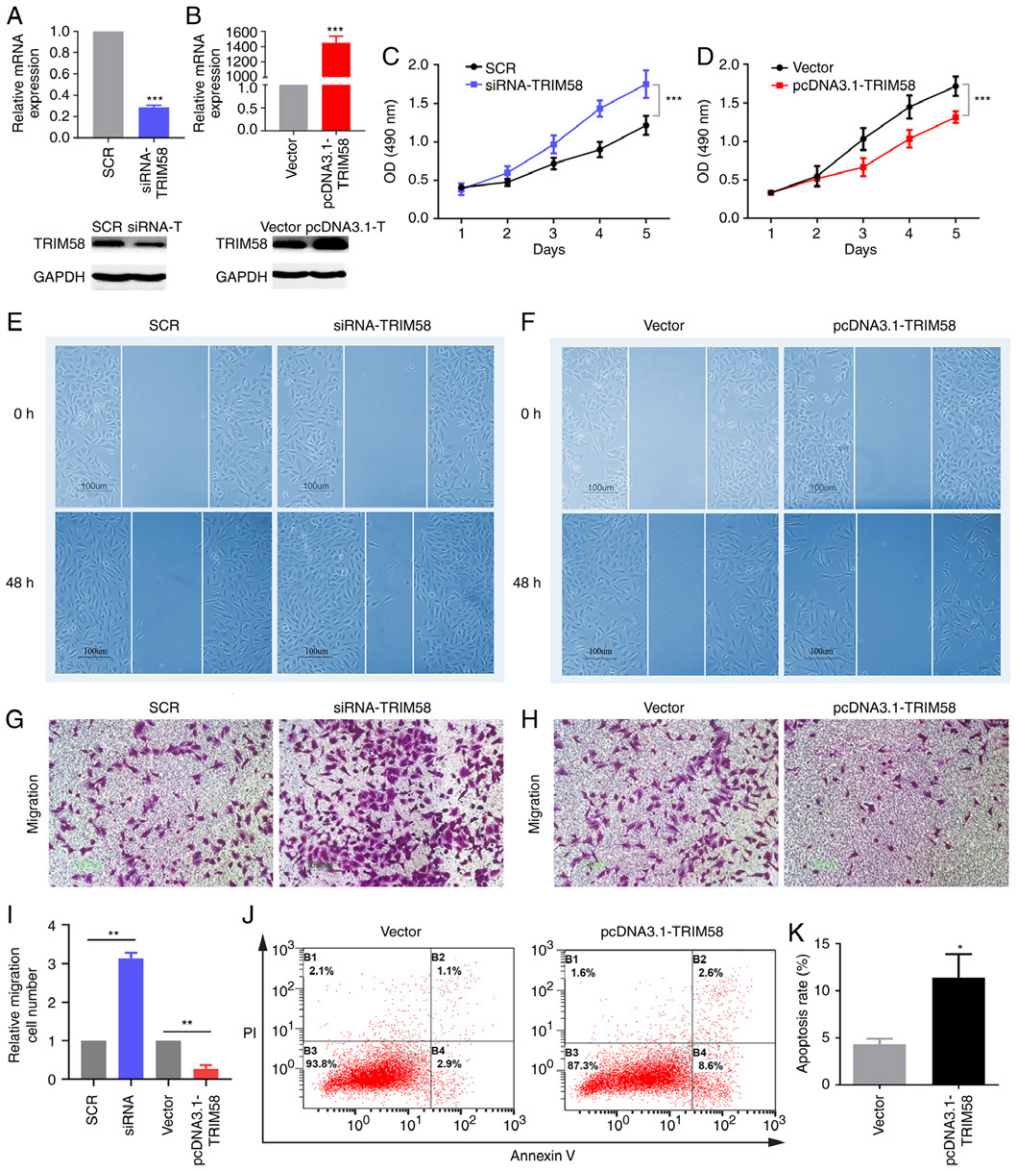
TRIM58 inhibits the malignant phenotypes of lung cancer cells. To evaluate the molecular functions of TRIM58 in lung cancer, loss-of-function and gain-of-function assays were conducted in A549 cell line. A series of transfection experiments were performed. (A and B) siRNA and plasmids effectively regulated the expression of TRIM58 in the A549 cell line. mRNA and protein expression of TRIM58 were detected by RT-qPCR and western blotting, respectively. (C and D) MTS assay was used to assess cell proliferation. Cell migration was assessed using the (E and F) wound healing assay and (G, H and I) transwell assay. (J and K) Flow cytometry was used to verify cell apoptosis. *P<0.05, **P<0.01 and ***P<0.001. SCR, scrambled negative control; si, small interfering; vector, pcDNA3.1; PI, propidium iodide; TRIM58, tripartite motif containing 58; OD, optical density. All these experiments are compared between the experimental group and the control group.

To evaluate the molecular functions of TRIM58 in lung cancer, loss-of-function and gain-of-function assays were conducted with the A549 cell line. The effects of silencing and overexpressing TRIM58 on the malignant phenotype were detected. Firstly, the expression of TRIM58 in A549 cells was silenced using siRNA. siRNA-TRIM58 was constructed using scramble siRNA as a negative control (Fig. 3A). The results demonstrated that compared with the control group, the siRNA-TRIM58 group exhibited significantly downregulated expression of TRIM58 (Fig. 3A). The MTS assay indicated that the silencing of TRIM58 promoted the proliferation of lung cancer cells (Fig. 3C). An overexpression vector TRIM58-pcDNA3.1 was constructed in the present study and an empty pcDNA3.1 vector was used as a negative control. The expression level of pcDNA3.1-TRIM58 was significantly increased compared with that of the vector (control group) (Fig. 3B). TRIM58 overexpression inhibited cell proliferation (Fig. 3D). Subsequently, wound-healing and transwell assays were used to evaluate cell migration. Compared with the control group (scramble siRNA), TRIM58 silencing potently accelerated the migration of lung cancer cells (Fig. 3E, G and I), whereas overexpression of TRIM58 exerted the opposite effect (Fig. 3F, H and I). In addition, flow cytometry analysis demonstrated that TRIM58 overexpression promoted the apoptosis of lung cancer cells compared with empty pcDNA3.1 vector (Fig. 3J and K). In summary, the overexpression of TRIM58, a potential tumor suppressor gene inhibited cell proliferation and migration and promoted cell apoptosis.

#### Identification of TRIM58-associated signaling pathways in lung cancer

**Figure 4.**
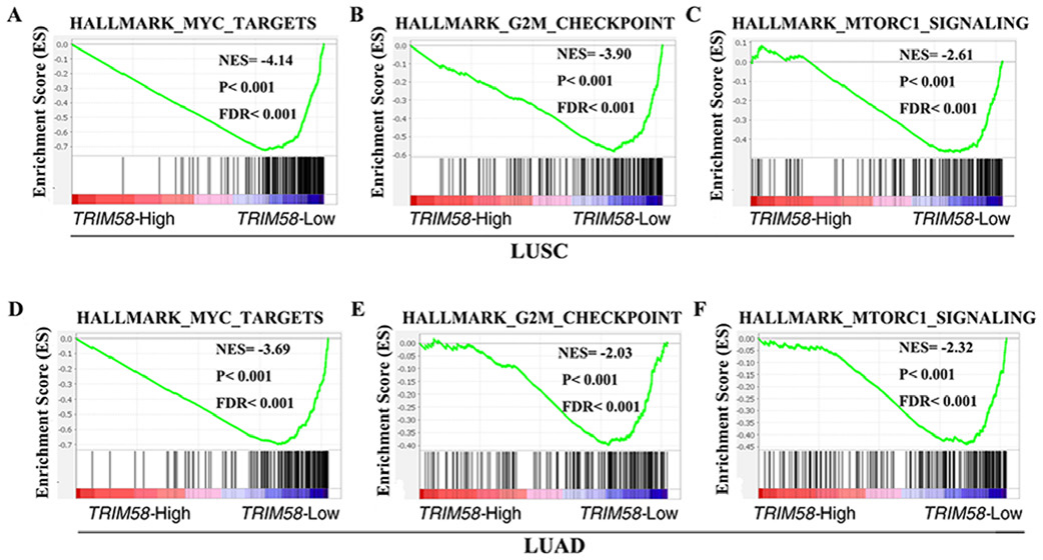
Identification of TRIM58-associated signaling pathway in lung cancer. GSEA demonstrated that TRIM58 was negatively associated with (A and D) MYC targets, (B and E) G2M checkpoints (C, and F) mTORC1 signaling pathway in LUSC and LUAD, respectively. TRIM58, tripartite motif containing 58; LUAD, lung adenocarcinoma; LUSC, lung squamous cell carcinoma; FDR, false discovery rate; mTORC1, mechanistic target of rapamycin complex 1; NES, normalized enrichment score.

To explore the molecular mechanisms by which TRIM58 contributes to lung cancer progression, GSEA was performed using mRNA expression data from the TCGA database. Several classic mechanisms of carcinogenesis, such as MYC targets [P<0.001; false discovery rate (FDR)<0.001; NES_LUSC_ =−4.14; P<0.001; FDR<0.001; NES_LUAD_=−3.69] (Fig. 4A and D) and G2M checkpoint-related genes (P<0.001; FDR<0.001; NES_LUSC_=−3.90; P<0.001; FDR<0.001; NES_LUAD_=−2.03) (Fig. 4B and E), were enriched in samples with low TRIM58 expression. It was also observed that TRIM58 was negatively correlated with the mTORC1 signaling pathway (P<0.001; FDR<0.001; NES_LUSC_=−2.61; P<0.001; FDR<0.001; NES_LUAD_=−2.32) (Fig. 4C and F), further indicating a tumor suppressor role of TRIM58.

#### Protein interaction and co-expression analysis

**Figure 5.**
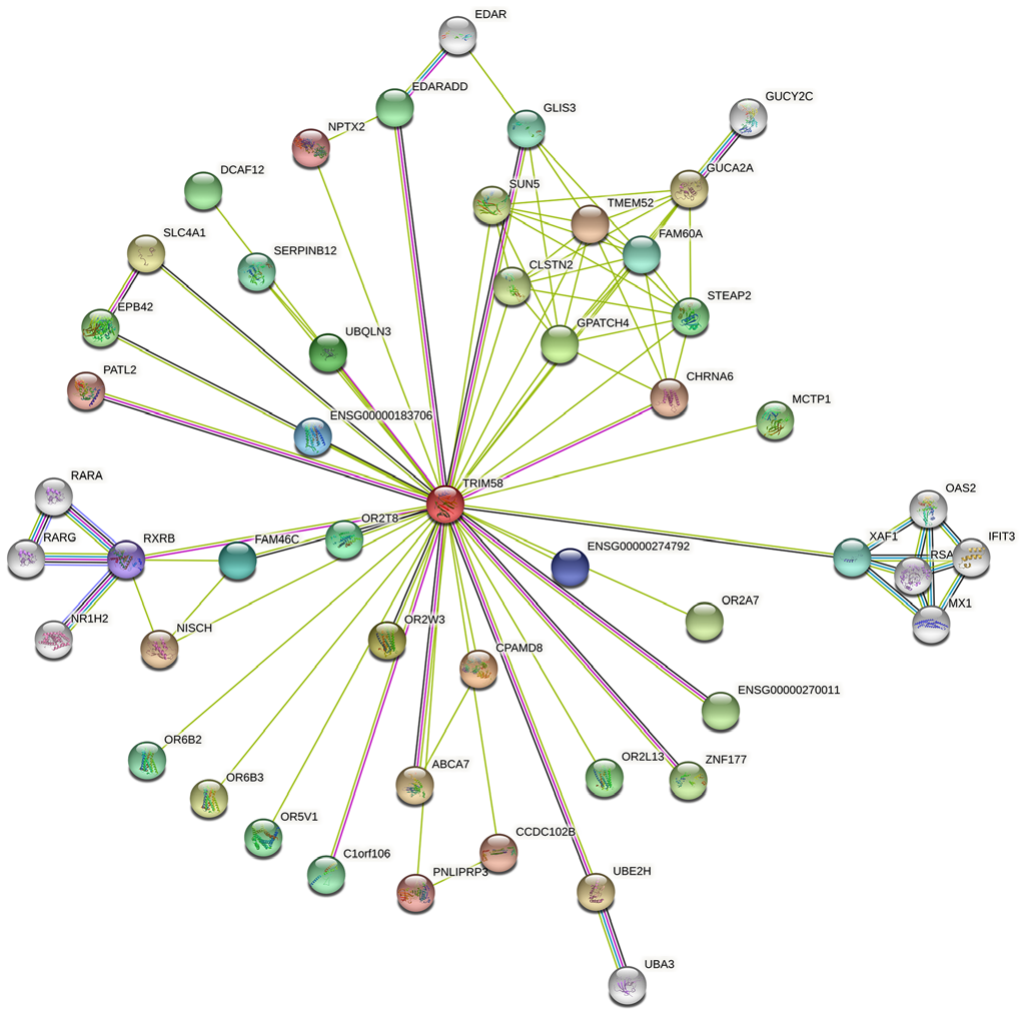
Protein-Protein Interaction network analysis. Using the STRING database, a series of proteins interacting with TRIM58 were identified. The minimum interaction score for this network is 0.4.

**Figure 6.**
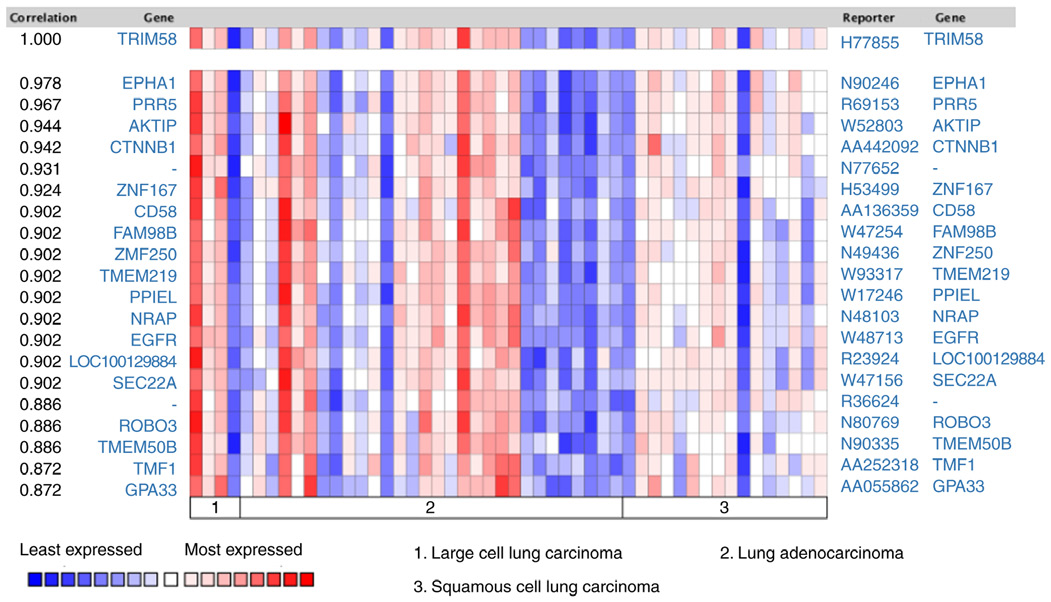
Co-expression analysis. The TRIM58 co-expression gene profile was identified by Oncomine. Blue squares represent least expressed, red squares represent most expressed.

Protein-Protein Interaction Network analysis was constructed using the STRING database (Fig. 5). A series of proteins interacting with TRIM58 were identified, such as CPAMD8 (C3 and PZP-like alpha-2-macroglobulin domain-containing protein 8), OR2W3 (Olfactory Receptor Family 2 Subfamily W Member 3), GPATCH4 (G Patch Domain-Containing Protein 4), UBQLN3 (Ubiquilin 3), OR2T8 (Olfactory Receptor Family 2 Subfamily T Member 8), FAM46C (Family with sequence similarity 46 member C) and RXRB (Retinoid X Receptor Beta). Subsequently, co-expression analysis was performed on lung cancer samples from the Oncomine database. Tomida *et al* (38) revealed that EPHA1 (EPH Receptor A1) (r=0.978), PRR5 (Proline-Rich Protein 5) (r=0.967), AKTIP (AKT Interacting Protein) (r=0.944), CTNNB1 (Catenin β-1) (r=0.942), ZNF167 (Zinc Finger Protein 167) (r=0.924), CD58 (CD58 Antigen) (r=0.902), FAM98B (Family With Sequence Similarity 98 Member B) (r=0.902), ZNF250 (Zinc Finger Protein 250) (r=0.902), TMEM219 (Transmembrane Protein 219) (r=0.902) were co-expressed with TRIM58 (Fig. 6). These results suggested that TRIM58 closely interacts with numerous functional genes involved in lung cancer.

## Discussion

Previous studies have shown that epigenetic changes can be used as biomarkers for the detection of malignant tumors, such as hypermethylation of GSTP1 (Glutathione S-Transferase Pi 1) in prostate cancer (8,39). Abnormal hypermethylation in the promoter region of tumor suppressor gene RASSF1A (RAS associated domain family 1 A), repair gene MGMT, apoptosis-related genes EBF3 (Early B Cell Factor 3), cell cycle-related gene CDKN2B (Cyclin-dependent kinase inhibitor 2B) and other important genes often occurs in precancerous lesions or during the early carcinogenesis of tumors, inhibiting transcriptional activity and leading to tumor occurrence (40–42). Additionally, abnormal genome-wide hypomethylation resulting in genomic instability and oncogene activation can induce tumors (43).

DNA methylation is an early event occurring in tumors, providing a stable signal with high sensitivity and specificity (44). In the present study, high-throughput screening and independent validation demonstrated that TRIM58 was hypermethylated and downregulated in lung cancer compared to normal tissues. ROC analysis performed in the present study revealed that TRIM58 had a strong predictive value for the early diagnosis of lung cancer.

TRIM family proteins serve an important role in tumorigenesis (17). There are few reports of the molecular mechanism of TRIM58's regulatory role in lung cancer. The morbidity and mortality of lung cancer is very high and tumor metastasis is an important cause of treatment failure and death (5). *In vitro* and *in vivo* experiments (45) demonstrated that TRIM62 inhibits the metastasis of cervical cancer by inhibiting the c-Jun/Slug signaling pathway. Chen *et al* (46) demonstrated that TRIM62 as an oncogene, negatively regulates TGF-β-mediated epithelial mesenchymal transition, hence inhibiting tumor invasion and metastasis. As a member of the same subfamily of TRIM62 (C-IV-1), it was hypothesized that TRIM58 may regulate the malignant phenotype of cancer. To further evaluate the molecular functions of TRIM58 in lung cancer, loss-of-function and gain-of-function assays were conducted in the present study in lung cancer cells (A549 cells). The results indicated that TRIM58 was a novel tumor suppressor gene in lung cancer.

In addition, the present study attempted to explore the signaling pathways related to TRIM58 in lung cancer to understand the potential mechanisms by which TRM58 participates in the regulation of tumor progression. Gene set enrichment analysis revealed that TRIM58 expression was negatively correlated with MYC targets, G2M checkpoints and the mTORC1 (mechanistic target of rapamycin complex 1) signaling pathway. mTOR is a protein kinase that can regulate a large number of cellular processes, such as cell growth, proliferation and differentiation through the PI3K/AKT/mTOR pathway (47). Among mTOR proteins, mTORC1 is frequently activated in human cancers and targeting mTORC1 signaling is a promising strategy for tumor therapy (48). In addition, MYC and G2M checkpoints are classic tumor-promoting signaling pathways (49). Hence, the results of the present study indicate that TRIM58 may serve an anticancer role by inhibiting the signaling pathways of mTORC1.

In conclusion, through DNA methylation-based profiling screening, the present study demonstrated that TRIM58 methylation has promise as a biomarker for the early diagnosis of lung cancer. Cell experiments confirmed the role of TRIM58 as a tumor suppressor gene in lung cancer and overexpression of TRIM58 inhibited the malignant phenotype of tumors. Gene set enrichment analysis revealed that TRIM58 expression was negatively correlated with the mTORC1 signaling pathway. Future studies are needed to further explore the specific regulatory mechanisms to provide new targets for the early diagnosis and effective treatment of lung cancer.

## Figures and Tables

**Table I.** Basic information of 55 differential methylation probes.

Ilmn ID (Probes)	Gene Symbol	Genbank Accession	Annotation
cg08572611	ACTL6B	NM_016188.3	Actin-like 6B
cg10235817	ADRA2C	NM_000683.3	Adrenoceptor α 2C
cg17619823	ADRB3	NM_000025.1	Adrenoceptor β 3
cg17525406	AJAP1	NM_018836.2	Adherens Junctions Associated Protein 1
cg20959866	AJAP1	NM_018836.2	Adherens Junctions Associated Protein 1
cg12111714	ATP8A2	NM_016529.3	ATPase Phospholipid Transporting 8A2
cg05890484	BHMT	NM_001713.1	Betaine-Homocysteine S-Methyltransferase
cg14419187	C2orf21	NM_182587.1	Unc-80 Homolog, NALCN Channel Complex Subunit
cg03544320	CRMP1	NM_001313.3	Collapsin Response Mediator Protein 1
cg09229912	CUTL2	NM_015267.1	Cut Like Homeobox 2
cg10303487	DPYS	NM_001385.1	Dihydropyrimidinase
cg04048259	EDN3	NM_000114.2	Endothelin 3
cg00027083	EPB41L3	NM_012307.2	Erythrocyte Membrane Protein Band 4.1 Like 3
cg08575537	EPO	NM_000799.2	Erythropoietin
cg20723355	FBXO39	NM_153230.1	F-Box Protein 39
cg19831575	FGF4	NM_002007.1	Fibroblast Growth Factor 4
cg02757432	GPR26	NM_153442.1	G Protein-Coupled Receptor 26
cg06722633	GRIK3	NM_000831.2	Glutamate Ionotropic Receptor Kainate Type Subunit 3
cg14859460	GRM6	NM_000843.2	Glutamate Metabotropic Receptor 6
cg26609631	GSH1	NM_145657.1	GS Homeobox 1
cg10883303	HOXA13	NM_000522.2	Homeobox A13
cg26069745	HOXA2	NM_006735.3	Homeobox A2
cg01354473	HOXA9	NM_152739.2	Homeobox A9
cg01381846	HOXA9	NM_152739.2	Homeobox A9
cg26521404	HOXA9	NM_152739.2	Homeobox A9
cg06760035	HOXB4	NM_024015.3	Homeobox B4
cg08089301	HOXB4	NM_024015.3	Homeobox B4
cg23130254	HOXD12	NM_021193.2	Homeobox D12
cg25574024	IGF2AS	NM_016412.1	Insulin-Like Growth Factor II, Antisense
cg23349790	IGSF21	NM_032880.2	Immunoglobin Superfamily Member 21
cg27409364	KCNC1	NM_004976.2	Potassium Voltage-Gated Channel Subfamily C Member 1
cg22660578	LHX1	NM_005568.2	LIM homeobox protein 1
cg04330449	NEUROG1	NT_034772.5	Neurogenin 1
cg22881914	NID2	NM_007361.2	Nidogen 2
cg08441806	NKX6-2	NM_177400.1	NK6 Transcription Factor Related, Locus 2
cg24194775	NPR2	NM_000907.2	Natriuretic Peptide Receptor 2
cg00548268	NPTX2	NM_002523.1	Neuronal Pentraxin 2
cg12799895	NPTX2	NM_002523.1	Neuronal Pentraxin 2
cg20291049	POU3F3	NM_006236.1	POU Domain Class 3, Transcription Factor 3
cg12374721	PRAC	NM_032391.2	PRAC1 Small Nuclear Protein
cg09516965	PTGDR	NM_000953.2	Prostaglandin D2 Receptor
cg08118311	SALL3	NM_171999.1	Spalt Like Transcription Factor 3
cg15191648	SALL3	NM_171999.1	Spalt Like Transcription Factor 3
cg02919422	SOX17	NM_022454.2	SRY-Box Transcription Factor 17
cg02164046	SST	NM_001048.3	Somatostatin
cg17586860	SSTR4	NM_001052.1	Somatostatin Receptor 4
cg25720804	TLX3	NM_021025.2	T Cell Leukemia Homeobox 3
cg14696396	TM6SF1	NM_023003.1	Transmembrane 6 Superfamily Member 1
cg01009664	TRH	NM_007117.1	Thyrotropin Releasing Hormone
cg07533148	TRIM58	NM_015431.2	Tripartite Motif Containing 58
cg07307078	TUBB6	NM_032525.1	Tubulin β 6
cg20616414	WNK2	NM_006648.3	WNK Lysine Deficient Protein Kinase 2
cg16638540	ZNF135	NM_003436.2	Zinc Finger Protein 135
cg03975694	ZNF540	NM_152606.2	Zinc Finger Protein 540
cg16731240	ZNF577	NM_032679.1	Zinc Finger Protein 577

## Data Availability

The datasets used and/or analyzed during the current study are available from the corresponding author on reasonable request.
